# Pulmonary embolism caused by ovarian vein thrombosis during cesarean section: a case report

**DOI:** 10.1186/s40981-017-0142-1

**Published:** 2018-01-05

**Authors:** Yutaka Oda, Michie Fujita, Chika Motohisa, Shinichi Nakata, Motoko Shimada, Ryushi Komatsu

**Affiliations:** 1 Department of Anesthesiology, Osaka City Juso Hospital, 2-12-27, Nonaka-kita, Yodogawa-ku, Osaka, 532-0034 Japan; 2 Department of Anesthesiology, Osaka City General Hospital, 2-13-22, Miyakojima Hondori, Miyakojima-ku, Osaka, 534-0021 Japan; 3 Department of Obstetrics and Gynecology, Osaka City Juso Hospital, 2-12-27, Nonaka-kita, Yodogawa-ku, Osaka, 532-0034 Japan; 4 Department of Cardiology, Osaka City Juso Hospital, 2-12-27, Nonaka-kita, Yodogawa-ku, Osaka, 532-0034 Japan

**Keywords:** Cesarean section, Ovarian vein thrombosis, Pulmonary embolism

## Abstract

**Background:**

Ovarian vein thrombosis is a rare complication of pregnancy. The representative complaints of patients with ovarian vein thrombosis are abdominal pain and fever. In some cases, however, fatal pulmonary embolism may develop. We report a case of pulmonary embolism presenting with severe hypotension and loss of consciousness during cesarean section possibly caused by ovarian vein thrombosis.

**Case presentation:**

A 25-year-old woman at 38 weeks 4 days of gestation was scheduled for repeat cesarean section. Her past history was unremarkable, and the progress of her pregnancy was uneventful. She did not experience any symptoms indicative of deep vein thrombosis. Cesarean section was performed under spinal anesthesia, and a healthy newborn was delivered. After removal of the placenta, she suddenly developed dyspnea, hypotension, and loss of consciousness with decreased peripheral oxygen saturation. Blood pressure, heart rate, and oxygen saturation recovered after tracheal intubation and mechanical ventilation with oxygen. Postoperative computed tomography revealed no abnormality in the brain or in the pulmonary artery, but a dilated right ovarian vein with thrombi, extending up to the inferior vena cava, was found. A diagnosis of pulmonary embolism caused by ovarian vein thrombosis was made, and heparin was administered. The tracheal tube was removed on the first postoperative day. Her postoperative course was uneventful, and she was discharged with no complications.

**Conclusion:**

Fatal pulmonary embolism might be caused by ovarian vein thrombosis during cesarean section. Careful and continuous observation of the patient after delivery and prompt treatment are important.

## Background

Pulmonary embolism (PE) is the leading cause of maternal mortality in the developed world [1, 2]. The incidence of PE increases during pregnancy and most often develops during the postpartum period [3]. Ovarian vein thrombosis, a rare complication during pregnancy, is a type of deep vein thrombosis that can lead to PE. The typical clinical presentation of ovarian vein thrombosis is lower abdominal pain and fever, but it can cause massive PE that leads to severe hypotension, syncope, and cardiac arrest in the postpartum period [4, 5]. There are several reports of PE occurring during cesarean section [6–9]. However, there have been no reports of PE caused by ovarian vein thrombosis during cesarean section. We report a case of a parturient who developed PE with respiratory arrest and loss of consciousness due to ovarian vein thrombosis during cesarean section.

## Case presentation

We have obtained written informed consent for publication of this case report from the patient. A 25-year-old woman (52.4 kg, 151 cm, gravida 2, para 2) at 38 weeks 4 days of gestation was scheduled for repeat cesarean section. Her past history was unremarkable, and she had no family history of venous thromboembolism or obesity. She underwent emergency cesarean section due to placental dysfunction at the age of 20, and elective cesarean section at the age of 22. Both procedures were performed uneventfully under spinal anesthesia, and oxytocin 5 units was intravenously administered as a uterotonic. The progress of the current pregnancy was uneventful. There was no abnormal bleeding or lower abdominal or leg pain indicative of deep vein thrombosis.

Preoperative electrocardiogram and chest X-ray were normal. The results of blood tests were unremarkable except for an increased serum d-dimer of 2.2 μg/ml. Blood pressure was 105/51 mmHg, heart rate was 54/min with sinus rhythm, and peripheral oxygen saturation was 98% at the time of entering the operating room. Spinal anesthesia was performed with 0.5% hyperbaric bupivacaine 11 mg mixed with fentanyl 20 μg and morphine 0.1 mg using a 25-gauge Quincke needle at the L3–L4 interspace after gentle aspiration of the clear cerebrospinal fluid in the right decubitus position. Analgesia level was below the T6 bilaterally.

Surgery was started 14 min after the start of anesthesia, and a healthy infant (2700 g) was delivered 9 min later with Apgar scores of 9 and 9, at 1 and 5 min, respectively. Oxytocin 5 units dissolved in 100 ml saline was infused, and the placenta was removed manually from the uterus. The patient was alert, was oriented, and responded to the voice of the newborn. Approximately 3 min after removal of the placenta, she abruptly developed dyspnea and lost consciousness, and it rapidly progressed into respiratory arrest. Blood pressure decreased to 48/29 mmHg, heart rate was 51/min, and peripheral oxygen saturation was 79%. Bag-mask ventilation with oxygen was immediately started, and the trachea was intubated after administration of propofol 50 mg, fentanyl 80 μg, and rocuronium 50 mg. Clear and bilaterally equal lung sounds were confirmed, and the end-tidal carbon dioxide tension was 35 mmHg. Peripheral oxygen saturation promptly returned to 100%, and blood pressure and heart rate increased to 105/54 mmHg and 111/min within 2 min without medications. Anesthesia was maintained with propofol 6 mg/kg/h until the end of surgery. There was no abnormal bleeding, and uterine contraction was good.

**Fig. 1:**
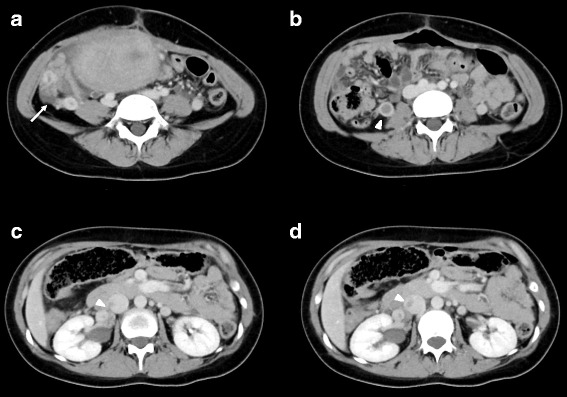
Computed tomography of the right ovarian vein and the inferior vena cava. The right ovary (white arrow) (**a**). A dilated right ovarian vein (18 × 19 mm) with a hypo-dense filling defect (white arrow head) (**b**). The right ovarian vein draining into the inferior vena cava (white arrow head) (**c**). The inferior vena cava with a hypo-dense filling defect indicating thrombi (white arrow head) (**d**)

The duration of surgery was 45 min with a total blood loss of 402 g including amniotic fluid and a urine output of 160 ml. Postoperative contrast-enhanced computed tomography (CT) revealed no abnormality in the brain or in the pulmonary arteries but a dilated right ovarian vein (18 × 19 mm) with a hypo-dense filling defect indicated the presence of thrombosis that extended up to the inferior vena cava (Fig. 1). She was transported to the high care unit (HCU) under sedation with propofol with tracheal intubation and mechanically ventilated overnight. Transthoracic echocardiographic examination revealed no abnormal findings, such as thrombi, dilated right ventricle, regurgitation of the tricuspid valve, or a shift of the interventricular septum in the heart.

A blood test performed immediately after entering the HCU revealed serum FDP was 390 μg/ml and d-dimer was 148.4 μg/ml. Other coagulation parameters such as fibrinogen, antithrombin III, and prothrombin time were within normal ranges. Based on the clinical presentation, CT image, and remarkably increased serum FDP and d-dimer levels, a diagnosis of PE was made. Heparin 3000 units was intravenously administered, and a continuous infusion of 10,000 units/day was started. The tracheal tube was removed after confirming stable hemodynamics and her prompt response to verbal commands on the first postoperative day, and she was discharged from the HCU. Oral administration of rivaroxaban 30 mg/day was started after stopping the infusion of heparin. Serum d-dimer decreased to 8.4 and 3.0 μg/ml on the first and second postoperative days, respectively. On the eighth postoperative day, CT examination detected the remaining thrombi in the right ovarian vein and her serum d-dimer was 1.5 μg/ml. She was discharged with the baby, and the remaining postoperative course was uneventful.

### Discussion

The diagnosis of maternal PE has been made based upon findings such as diminished lung perfusion as assessed using ventilation-perfusion scintigraphy and thrombi detected with CT pulmonary angiography and ultrasonography. These diagnostic tools are used with the same criteria to diagnose non-maternal PE [3, 10, 11]. However, there have been no validated criteria for diagnosing maternal PE. Despite a lack of these symptoms in the present case, we made a diagnosis of PE based on sudden onset of respiratory arrest, hypotension, and loss of consciousness together with ovarian vein thrombosis. This diagnosis facilitated the initiation of anticoagulant therapy. It is important to note that the diagnosis of possible PE during pregnancy can be made based exclusively upon signs and symptoms consistent with it [3].

The loss of consciousness would suggest a sudden obstruction of the most proximal pulmonary arteries, resulting in a transient decrease in cardiac output [12]. Remarkably increased plasma FDP and d-dimer levels after surgery further suggest the rapid progression of blood coagulation and fibrinolysis. Prompt hemodynamic recovery and the lack of abnormal findings on CT pulmonary angiography and transthoracic echocardiography rule out other etiologies such as air or fat embolism, acute coronary spasm, or aortic dissection and suggest that the thrombus lodged distantly in the pulmonary vasculature. Clinical presentation of amniotic fluid embolism also includes sudden hypoxia and hypotension, as observed in the present case. However, coagulopathy accompanied with decreased fibrinogen and platelet count is accompanied in most of those patients [13, 14]. A lack of coagulopathy would strongly suggest that sudden changes of hemodynamic and respiratory conditions in the present case would have been caused by thrombosis, not by amniotic fluid embolism.

Pregnant women are at an increased risk of venous thromboembolism including deep vein thrombosis and PE. The elements of Virchow’s triad that contribute to thrombosis: venous stasis, vascular damage, and hypercoagulopathy, are all present during pregnancy and the postpartum period [10]. According to a 30-year population-based study in the USA, the incidence of PE is 47.9 per 100,000 women-years and is more than 15 times higher in the first three postpartum months than during pregnancy [3]. The incidence also significantly increases for postpartum women in older age groups (≥ 35 years) compared with younger women [3].

Early detection and adequate intervention is crucially important in order to prevent PE. In our case, however, there were no symptoms, such as swelling and pain in the leg and lower abdomen, that suggested the presence of deep vein thrombosis. Although Doppler ultrasonography has been widely used for this purpose, postoperative contrast-enhanced CT detected no thrombi in the leg, suggesting that it would not have been detected before surgery. Serum d-dimer levels increase to 489–2217 ng/ml (5th–95th percentile) in the last trimester [15] and result in poor specificity for diagnosing PE [10]. An increased plasma d-dimer alone does not suggest PE or, because of the teratogenic and oncogenic effects of radiation, prompt us to use diagnostic imaging preoperatively.

Ovarian vein thrombosis is a rare but serious postpartum complication with an incidence between 0.05 to 0.18% [4, 5]. In 90% of cases, the thrombus occurs in the right ovarian vein. This is partly due to the dextrotorsion of the puerperal enlarged uterus which might compress the ovarian vein [5]. The most common symptoms of ovarian vein thrombosis are lower abdominal pain and fever, but it may progress to involve the vena cava and induces life-threatening pulmonary embolism as occurred in the present case [4]. A thrombus was formed asymptomatically during our patient’s pregnancy, and following the release of the compression on the ovarian vein by gravid uterus and intrauterine maneuver, the more proximal parts of the thrombus disengaged and lodged in the pulmonary vasculature. There are several reports describing cardiac arrest caused by PE due to deep vein thrombosis during cesarean section after delivery [6, 7].

Anesthesiologists should carefully observe patients after delivery as well as immediately after spinal anesthesia because of the unpredictable cardiorespiratory collapse that may occur in this period due to conditions such as PE and anaphylactic reactions [16]. Immediate tracheal intubation, medications including adrenaline to restore hemodynamic state, and blood test for examining coagulopathy are strongly recommended if such conditions occur during cesarean section. Measurement of blood tryptase is also helpful if anaphylaxis is suspected [17]. Intraoperative transesophageal echocardiography would be useful for differential diagnosis of pulmonary embolism and other conditions such as acute coronary syndrome and aortic dissection.

## Conclusions

Here, we reported the case of PE during cesarean section in which the patient developed sudden onset of dyspnea and loss of consciousness with decreased blood pressure, heart rate, and peripheral oxygen saturation. Her cardiorespiratory signs spontaneously recovered after tracheal intubation and ventilation with oxygen. Although there were no abnormal findings in the pulmonary arteries or in the heart, postoperative CT pulmonary angiography revealed thrombi in the right ovarian vein that progressed to the inferior vena cava and would have been responsible for PE.

## Abbreviations

CT: Computed tomography
PE: Pulmonary embolism
